# Overview of Chios Mastic Gum (*Pistacia lentiscus*) Effects on Human Health

**DOI:** 10.3390/nu14030590

**Published:** 2022-01-28

**Authors:** Stergios Soulaidopoulos, Aikaterini Tsiogka, Christina Chrysohoou, Emilia Lazarou, Konstantinos Aznaouridis, Ioannis Doundoulakis, Dimitra Tyrovola, Dimitris Tousoulis, Konstantinos Tsioufis, Charalambos Vlachopoulos, George Lazaros

**Affiliations:** 1 First Cardiology Department, Hippokration General Hospital, School of Medicine, National and Kapodistrian University of Athens, 11527 Athens, Greece; soulaidopoulos@hotmail.com (S.S.); chrysohoou@usa.net (C.C.); emilazarou@gmail.com (E.L.); conazna@yahoo.com (K.A.); doudougiannis@gmail.com (I.D.); dimitra.tyrovola@gmail.com (D.T.); drtousoulis@hotmail.com (D.T.); ktsioufis@gmail.com (K.T.); cvlachop@otenet.gr (C.V.); 2 First Department of Dermatology-Venereology, Andreas Sygros Hospital, School of Medicine, National and Kapodistrian University of Athens, 16121 Athens, Greece; katerina_sse@hotmail.com

**Keywords:** mastic gum, mastiha, *Pistacia lentiscus*, anti-inflammatory effect, oxidative stress, inflammatory bowel disease, oxidized LDL

## Abstract

Despite the remarkable development of the medical industry in the current era, herbal products with therapeutic potentials arise as attractive alternative treatments. Consequently, Chios mastiha, a natural, aromatic resin obtained from the trunk and brunches of the mastic tree, has recently gained increasing scientific interest due to its multiple beneficial actions. Chios mastiha is being exclusively produced on the southern part of Chios, a Greek island situated in the northern Aegean Sea, and its therapeutic properties have been known since Greek antiquity. There is now substantial evidence to suggest that mastiha demonstrates a plethora of favorable effects, mainly attributed to the anti-inflammatory and anti-oxidative properties of its components. The main use of mastiha nowadays, however, is for the production of natural chewing gum, although an approval by the European Medicines Agency for mild dyspeptic disorders and for inflammations of the skin has been given. The aim of this article is to summarize the most important data about the therapeutic actions of Chios mastiha and discuss future fields for its medical application.

## 1. Introduction

Mastiha is a natural, aromatic resin obtained from the trunk and brunches of the mastic tree (*Pistacia lentiscus* L. *var latifolius Coss* or *Pistacia lentiscus var. Chia*). It is also known as Chios mastic gum, being exclusively produced on the southern part of Chios, a Greek island situated in the northern Aegean Sea. Although *Pistacia* species are widespread across the Mediterranean basin and the surrounding regions, mastiha is produced only by the mastic trees grown on the island of Chios, where 24 villages (Mastichochoria in Greek) maintain the region’s cultural heritage and participate in the resin’s production. The life cycle of most trees is about 100 years, and the yearly production ranges from 60 to 250 g per tree. After the induction of small cuts on the tree’s bark and branches, the resin is produced and collected in “tears” or droplets, which are initially translucent white or pale-yellow, acquiring a more yellowish and opaque color as they age [1].

Mastiha has been harvested for more than 2500 years, and it is already known in Greek antiquity for its therapeutic properties. The famous physician of the Greek classical era Hippocrates used mastiha to treat gastrointestinal disorders. During the Roman and Byzantine periods, the recognition of its healing effects led to the development of the trade of mastiha, which constitutes one of the monopolies managed by the Sultan in the years of the Ottoman Empire. In recent era, despite the remarkable development of the medical industry, mastiha still maintains a unique position among herbal treatments. Recognizing its therapeutic value, the European Medicines Agency (EMA) has included Chios mastiha in the category of traditional herbal medicines, approving the use of mastiha for mild dyspeptic disorders and for inflammation or minor lesions of the skin [2]. The main use of mastiha nowadays, however, is for the production of the natural chewing gum of Chios with a characteristic bitter taste, for dental and culinary purposes, and as a food ingredient in the Mediterranean region.

This therapeutic potential of mastiha can be attributed to the action of a variety of compounds with anti-inflammatory and antioxidant properties [3]. It represents a concentrated, natural source of terpenes, phenolic compounds, phytosterols, arabino-galactanes proteins, and natural polymers, along with volatile and aromatic ingredients, displaying a plethora of bioactive effects (Table 1) [4,5]. Nevertheless, the bioaccessibility and bioavailability of these ingredients is not fully understood, while the precise mechanisms underlying mastiha’s beneficial effects remain yet largely unknown. In this context, the aim of this article is to summarize the most important therapeutic actions of Chios mastiha and discuss scientific evidence supporting the expansion of its medical application.

## 2. Anti-Inflammatory Properties

Inflammation constitutes the common pathogenetic substrate for many acute and chronic diseases. It represents the human’s immune system response to various stimuli, such as pathogens, toxic compounds and damaged cells, regulated by a variety of endogenous factors, including cytokines, growth factors and activated cells [6]. There are two types of inflammation, acute and chronic. While acute inflammation usually refers to a short-term activation of the immune system as a response to an external trigger, chronic inflammation may occur in the absence of any specific stimuli, resulting in the development of many chronic diseases. It is now well established that several chronic disorders, such as connective tissue and inflammatory bowel diseases, diabetes mellitus, cancer and cardiovascular disorders share, to a lesser or greater degree, common pathogenetic mechanisms involving inflammation [7].

There is now an emerging body of evidence to support the anti-inflammatory activity of Chios mastiha. This anti-inflammatory action seems to be performed via the inhibition of the production of pro-inflammatory substances. In particular, administration of both solid and liquid types of mastiha seems to inhibit prostaglandin secretion along with inducible nitric oxide synthase (iNOS) and cyclooxygenase (COX)-2 expression by macrophages at both protein and mRNA level in animal experimental models [8]. In vitro, mastiha blocks the expression of the adhesion molecules VCAM-1 and ICAM-1 by TNF-alpha-stimulated human aortic endothelial cells, thereby interfering in endothelial activation that is recognized as the primary event of the atherosclerotic process [9].

Data derived by human studies are also in the same direction (Table 2). In a small clinical study including 10 patients with mild or moderately active Crohn’s disease recruited to treatment with mastic caps for 4 weeks (2.2 g/day), a significant decrease in the activity index of the disease and the plasma levels of interleukin-6 and CRP compared to baseline was observed, while no significant side effects were reported [10]. In the same patient cohort, a remarkable reduction in TNF-a secretion following treatment with mastic caps was later reported, suggesting an additional inhibitory mechanism of monocyte chemotaxis, thus providing more support to the role of mastiha as immune system regulator [11].

**Figure 1:**
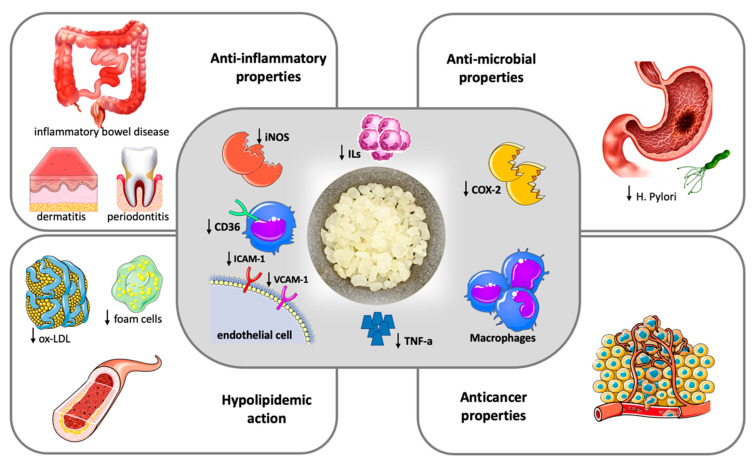
Therapeutic potentials of Chios Mastic.

Based on the findings of this pilot study, in 2019, Papada et al. designed and performed a randomized controlled trial to further investigate the impact of mastiha on patients with inflammatory bowel disease. A total of 60 patients were randomly assigned to receive either mastiha (2.8 g/day) or placebo for 3 months adjunct to standard medication. Patients treated with mastiha had a significant decrease in faecal lysozyme compared to patients on placebo, indicative of lower disease activity. In addition to this, a significant improvement in Inflammatory Bowel Disease Questionnaire scores reflecting a beneficial effect on patients’ quality of life was observed in the mastiha arm compared to the baseline [12]. When the same protocol was applied to 68 patients with inactive inflammatory bowel disease for 6 months, in contrast to controls, patients allocated to mastiha as add-on treatment to standard medication presented no increase in interleukin-6 or in faecal biomarkers calprotectin and lactoferrin, which are neutrophil-derived proteins whose concentrations typically rise in patients with gastrointestinal mucosal inflammation [14]. Recent data support that mastiha treatment interferes in the regulation of Th17 cell function and differentiation, resulting in increased serum levels of interleukin-17A that is considered to play a protective role in the development and relapse of inflammatory bowel disease [15]. Figure 1 summarizes the most important pathogenetic and clinical effects of mastic.

Chios mastic exerts anti-inflammatory and antioxidative properties (central frame). Anti-inflammatory action is attributed to the inhibition of inducible nitric oxide synthase (iNOS) and cyclooxygenase (COX)-2 expression by macrophages and the blockage of the expression of the adhesion molecules VCAM-1 and ICAM-1 by TNF-alpha stimulated endothelial cells, ultimately resulting in reduced Tumor Necrosis-alpha (TNA-a) and inflammatory interleukins (ILs) production. The antioxidative properties are mainly driven by a downregulation of CD36 expression in macrophages along with an increase in the intracellular antioxidant glutathione levels. The clinical effects of mastic (outer frames) represent the result of anti-inflammatory and anti-oxidant action and include a hypolipidemic action with a decrease in oxidized-LDL (ox-LDL) particles and foam cell formation, beneficial effects in inflammatory bowel disease, dermatitis and periodontal inflammation, antimicrobial and anticancer properties.

Mastic demonstrates a protective effect on intestinal epithelial cells, largely determined by its anti-inflammatory and antioxidant properties [25]. This action has been more thoroughly investigated in inflammatory bowel diseases, where mastic has been found to decrease the cytokines tumor necrosis factor-a, malonaldehyde, intercellular adhesion molecule-1 (ICAM-1) and interleukin -6, -8 and -10, both in preclinical and clinical studies; thus, efficiently inhibiting intestinal damage [26,27]. In addition, mastiha supplementation promotes a partial but respectable recovery of microbial diversity, acting as a natural probiotic factor [28]. A randomized controlled trial including 148 subjects showed that mastic, at a dose of 350 mg three times daily, significantly improved symptoms related with functional dyspepsia after 3 weeks of treatment [16].

It has also been found that mastic reduces liver enzymes and improves hepatic steatosis and collagen content in experimental models with non-alcoholic fatty liver disease (NAFLD) [28]. Considering that oxidation and inflammation dominate the pathogenetic substrate of NAFLD, the MAST4HEALTH randomized clinical trial reported a significant improvement in total antioxidant status of obese patients with NAFLD treated with mastiha for a 6 month period [17]. Several mastic compounds, including oleanonic acid, oleanolic acid and gallic acid act as modulators of peroxisome proliferator-activated receptors (PPARs), which are recognized as regulators of glucose and lipid metabolism, inflammation and fibrosis progression in the liver, playing a crucial role in the development of NAFLD [29].

There is an accumulating body of evidence suggesting that the topical application of mastic ointment attenuates inflammatory and/or pruritic responses in animal experimental models of allergic dermatitis [30]. This action is once again attributed to the anti-inflammatory properties of mastic and, particularly, to the drastic reduction of cytokine production. Clinical data derived from randomized controlled trials also suggest that mastic exhibits a beneficial effect on wound healing. Higher healing rates of episiotomy wound healing were observed in 73 women who were treated for three days postpartum with mastic oleoresin, which was administered through smoking of the wound [18]. These data are consistent with findings provided by animal studies that demonstrate a favorable action of mastic oil on the healing of wounds caused by laser burns [31].

The aforementioned data undoubtedly support that mastic possesses anti-inflammatory properties. On the other hand, with the exception of inflammatory bowel disorders, data regarding the potential anti-inflammatory effect of mastiha on other systemic inflammatory disorders are scarce. In these terms, large-scale, well-designed clinical trials involving patients with common inflammatory disorders are yet to be performed in order to establish the anti-inflammatory function of mastiha.

## 3. Anti-Oxidant Properties

Oxidative stress denotes an imbalance between the production of reactive oxygen species (ROS) and the antioxidant defense of a biological system [32]. Free radicals of oxygen produced by the cellular metabolism play an integral role in the modulation of cell signaling, differentiation and proliferation.

Excessive production of ROS may, however, cause oxidative stress, which is responsible for harmful changes on DNA, RNA protein and lipids, and associated with an increased risk for several chronic and systemic disorders—namely cardiovascular disease, systemic inflammatory disorders and cancer [33]. To that end, natural antioxidants have gained substantial scientific interest, driven by their numerous health benefits.

The oxidization of LDL is representative of changes induced by oxidative stress. After multiple modifications taking place on LDL particles, the oxidized LDL acquire atherogenic and pro-inflammatory properties, drastically contributing to the development of atherosclerosis [34]. Investigating the antioxidant potential of several gums and resins in vitro, Andrikopoulos et al. demonstrated that mastiha was the most effective one in preventing human LDL oxidation [35]. This action was mainly attributed to the hydromethanolic component of mastiha, with triterpenes and hydroxynaphthoquinones demonstrating LDL protective activity as well.

Mastiha attenuates cellular superoxide production by downregulating NADPH oxidase through the inhibition of protein kinase C pathways, a process that is possibly triggered by TNF-a, again underlining the close interaction between inflammation and oxidative stress [19]. Protein kinase C is known to hold a key position in a variety of cellular signaling pathways, being reversibly modulated by ROS owing to its unique structural feature that is susceptible to oxidative modification [36]. Inhibition of such pathways by mastiha, particularly by its triterpenes, leads to an increase in the intracellular antioxidant glutathione levels along in macrophages along with a downregulation of CD36 expression [37]. The latter are recognized as the oxLDL receptors in macrophages and keep a central role in the foam cell formation atherogenesis, upregulated in the presence of oxLDL and interleukin-4 [38].

## 4. Anti-Atherogenic Properties

The anti-atherogenic effects of Chios mastic are associated with its anti-inflammatory and antioxidant properties. As aforementioned, beyond intracellular antioxidant glutathione enhancement, it is suggested that mastic triterpenes exhibit their antioxidant effect by downregulating CD36 expression in macrophages, thus preventing oxLDL uptake that promotes the formation and accumulation of foam cells at sites of vascular endothelial dysfunction in both early and late steps of atherosclerosis. In animal experimental models, mastic treatment has been found to possess anti-ischemic properties, leading to a reduction in infarct size [39]. However, clinical evidence on the anti-atherogenic or cardioprotective effects of mastic is limited, and mainly derived from studies assessing surrogate markers of atherosclerosis.

A remarkable reduction in lipid levels was demonstrated in a clinical trial including 133 patients, who were randomly assigned to receive either high-dose (5 g/daily) or low-dose (mastic solution) mastiha treatment. More specifically, patients on high-dose mastic presented a significant decrease in serum total cholesterol, LDL, total cholesterol/HDL ratio, lipoprotein a, apolipoprotein A-1 and apolipoprotein B levels after an 18-month treatment period. Moreover, a decrease in liver enzyme values in the same group was observed [40]. Similar findings were reported in 60 patients with inflammatory bowel disease, randomized to either mastic treatment (2.8 g/day) or placebo for 3 months while receiving standard treatment for their disease. Treatment with mastic was associated to a significant reduction in oxLDL levels along with oxLDL/LDL and oxLDL/HDL ratios [13].

The ‘Chios-Mastiha’ trial included 156 patients with total cholesterol values > 200 mg/dl who were randomly allocated to placebo, 1 g/day of crude mastic (total mastic group), 1 g/day of polymer free mastic or 2 g/day of powder mastic. Of note, after 8 weeks of treatment, only patients treated with 1 g of crude mastic per day exhibited a significant reduction in total cholesterol values of about 11.5 mg/dl compared to baseline, with this effect being more pronounced in overweight and obese patients. This was accompanied by a decrease in free plasma glucose levels of about 4.5 mg/dl. Nonetheless, these findings should be interpreted with caution, given that no effects on LDL, HDL, triglycerides or CRP were observed [20]. In a different concept, compared to placebo, mastiha treatment exerted favorable effects on peripheral and aortic hemodynamics, as assessed by non-invasive aortic blood pressure measurements and aortic augmentation index among 13 hypertensive patients [21].

These data imply that mastiha may display cardioprotective effects driven by its lipid-lowering action. For the moment, there are a lack of studies with hard cardiovascular end-points, which are expected to shed more light on the cardioprotective potential of mastiha in the future.

## 5. Anticancer Properties

There are several limitations in the assessment of the potential contribution of diet supplements as anticancer agents in vivo, owing to the high heterogenity regarding the clinical course and the underlying signaling of the various types of cancer along with the common co-administration of cytotoxic drugs. On the other hand, the use of food-related components as an add-on weapon to specific types of cancer attracts substantial scientific interest, aiming to enhance the efficacy and the reduce toxicity of conventional treatments.

Data regarding the anticancer activity of Chios mastic derive from a deteriorated number of in vitro and animal experimental studies with otherwise promising findings. More specifically, the hexane extract of mastic induces the apoptosis of HCT116 human colon cancer cells in vitro through a caspase-related mechanism [41,42]. Mastic attenuates the expression and function of the androgen receptor, which has a central role in the development and progression of prostate cancer [43]. In addition to this, through the suppression of NF-κΒ activity and the NF-kB signaling pathway, mastic inhibits the cell cycle progression in prostate cancer cells [44]. An antiproliferative and proapoptotic activity of mastic oil was also demonstrated on human K562 leukemia cells in vitro [45]. It was also found to prevent the vascular endothelial growth factor, hence decreasing microvessel formation from mouse melanoma cells both in vitro and in vivo [45].

These preliminary findings imply that mastic may exhibit an anticancer effect, which, nevertheless, is yet to be investigated in further in vivo studies.

## 6. Antibacterial Properties

Mastic is rather characterized by a wide spectrum of antimicrobial activity, which extends beyond the H. Pylori eradication that has already been mentioned. This has been known since at least 1995, when the addition of mastic oil In broth cultures was shown to effectively inhibit the growth of both Gram-positive and Gram-negative bacteria, including Staphylococcus aureus, Lactobacillus plantarum, Pseudomonas fragi and Salmonella enteritidis. In fact, the inhibitory effect was proportionate to the concentration of the added oil [46]. More recent studies have demonstrated a strong antimicrobial action of mastic against Streptococcus species and several other common pharyngeal and ear pathogens, either when administered alone or in combination with antibiotics [47]. Moreover, the effective inhibition of numerous periodontal pathogens, without exhibiting any cytotoxic effects towards cells of epithelial or mesenchymal origin, makes mastic gum an ideal antimicrobial factor for oral inflammatory diseases [48].

A small number of individual reports suggests an antifungal action of mastic. Of note, there is now evidence supporting the antimicrobial action of mastic against several clinical isolates of Candida species, which are frequently resistant to conventional antimicrobial treatments [49]. Different concentrations of mastic were also found to inhibit Trichomonas vaginalis multiplication, a protozoan parasite that causes trichomoniasis, a cosmopolitan sexually transmitted disease [50].

The antibacterial activity of mastic resin upon Helicobacter Pylori (H. Pylori) is probably the one that has been more adequately investigated, both in vitro and in clinical trials. H. Pylori infection is recognized as one of the main factors for gastritis, peptic ulcer disease and gastric cancer, with its treatment being of crucial importance for the management and prevention of prevalent digestive disorders [51]. H. Pylori eradication treatment usually consists of clarithromycin, with either amoxicillin or metronidazole, and a proton pump inhibitor administered for 7–14 days [52]. Given that eradication is achieved in less than 85% of treated patients due to resistance to antibiotics, there is an urgent need for alternative therapies that could efficiently contribute to the management of infectious diseases [53]. The first evidence suggesting that mastic has antibacterial activity against H. Pyloriwas published in the New England Journal of Medicine in 1998, providing a possible pathogenetic interpretation of the beneficial effect of mastic on gastric and duodenal ulcers that was already known [54].

In line with this report, mastic was shown to kill 90% of isolated H. Pylori strains at a concentration of 500 μg/mL [55]. This seems to be attributed to arabinogalactan proteins which inhibit neutrophil activation in the presence of H. pylori neutrophil-activating protein [22]. In a randomized clinical trial where different doses of mastic, either as single therapy or in combination with pantoprazole, were administered in patients with H. Pylori infection for 14 days, it was confirmed that mastic is sufficient in achieving H. Pylori eradication in vivo. Nevertheless, failure of the combination of mastic with pantoprazole to eradicate H. Pylori was observed, attributed to the hypothesis that most of the mastic substances require an acidic environment in the stomach to be effective against H. Pylori, a condition that is changed with proton pump inhibitors that increase gastric alkalinity [23]. Underlining the need for more randomized studies to be performed, Bebb et al. reported no effect on H. Pylori status as assessed by urea breath tests in eight patients treated with mastic [24].

## 7. Adverse Effects—Toxicity

Mastic gum consumption is generally considered safe, although the long-term safety has not been sufficiently investigated, and the maximum safe dose remains still unknown. Beyond some cases of allergic contact dermatitis following postoperative use of patches containing mastic, there are hardly any reports of remarkable side effects [56]. High doses of mastic have also been well tolerated in clinical trials, and no adverse effects have been recorded.

There are only specific reports of possible side effects of mastic originating from animal studies. High doses of mastic may induce renal histological alterations in rats while displaying cytotoxic effects on specific cell series [57]. A dose-related increase in liver weights along with unfavorable changes in several hematological and biochemical parameters was observed in rats treated with high doses of mastic for 13 weeks [58]. Investigating the modifying effects of mastic on rat liver carcinogenesis, another study reported an increase in parameters related to the formation of hepatic preneoplastic lesions after mastic administration in cancer bioassay models [59]. On the contrary, mastic showed an antihepatotoxic activity in carbon tetrachloride-intoxicated rats leading to a reduction in bilirubin levels and in the activity of alkaline phosphatase [60].

Both the beneficial and adverse effects of mastic described in previous paragraphs are likely dose-, time- and tissue-dependent. Nevertheless, more research focusing on tissue distribution and pharmacokinetics of mastic is needed in order to precisely determine the resin’s therapeutic and toxic doses.

## 8. Conclusions

Despite the great progress that has been made on human health and the remarkable ongoing development in medical products, there is an increasing interest, nowadays, for natural supplements that may exert beneficial health effects. In this context, the existing data suggest that Chios mastic possesses anti-inflammatory and anti-oxidative properties which could be utilized in the treatment of multiple disorders. Given the emerging antimicrobial resistance trends, the establishment of mastic’s antibacterial efficacy could support its introduction as adjunct therapy in the management of various infectious diseases. Moreover, the antilipidemic activity of mastic that has been observed prompts the conduction of clinical trials with cardiovascular endpoints to assess its possible value in the management of cardiovascular disorders. Another point that should be underlined is that, to date, no significant adverse effects associated with human consumption of mastic have been reported.

Overall, Chios mastic gathers many favorable properties that could justify its therapeutic use for a variety of human diseases. Most of the research data, however, derive from studies on animal experimental models or studies performed in vitro, while the number human studies in this direction are, for the moment, limited. There is, therefore, need for further clinical research in order to assess the therapeutic potential of mastic in different disorders and to unravel its complex mechanism of action.

## Figures and Tables

**Table 1:** Compounds of Chios mastic.

Compound
1,4-poly-β-myrcene
20(S)-3β-acetoxy-20-hydroxydammara-24-ene
3-oxo-28-norlup-20 (29)-ene
3β-hydroxy-28-norolean-12-ene
3-oxo-28-norolean-12-ene
3-oxo-dammara-20 (21),24-diene
(8R)-3-Oxo-8-hydroxy-polypoda13E,17E,21-triene
3-oxo-malabarica-14(26),17E,21-triene
3β-hydroxymalabarica-14(26),17E,21-triene
banillic acid
gallic acid trans-cinnamomic acid
isomasticadienonic acid
masticadienolic acid
moronic acid
oleanolic acid
oleanolic aldehyde
p-hydroxy-benzoic acid
p-hydroxy-phenylacetic acid
tirucallol
tyrosol

**Table 2:** Human studies assessing mastic’s effects.

Study	Design	Effect
Kaliora et al. [10]	10 pts with active CD and 8 healthy controls 2.2 g of mastic daily for 4 weeks	- Decrease in CD activity index - decrease in IL-6 and CRP - no effect in plasma TNF-a
Kaliora et al. [11]	10 pts with active CD and 8 healthy controls 2.2 g of mastic daily, 4 weeks	- Reduction of TNF-a secretion by mononuclear cells - increase in macrophage migration inhibitory factor
Papada et al. [12,13]	60 pts with IBD randomized to either 2.8 g of mastic daily for 3 months or placebo	- Improvement in IBDQ - Decrease in oxLDL - Decrease in plasma cysteine and faecal lysozyme
Papada et al. [14]	68 pts with IBD randomized to either 2.8 g of mastic daily for 6 months or placebo	- No impact on serum IL-6, faecal calprotectin and faecal lactoferrin
Amerikanou et al. [15]	129 pts with IBD—68 randomized to mastic group (2.8 g daily for 6 months for pts in remission and for 3 months for pts in relapse) and 61 to placebo	- Increase in IL-17A
Dabos et al. [16]	148 pts with functional dyspepsia randomized to either mastic 350 mg tid or placebo for 3 weeks	- Significant improvement of symptoms - (stomach pain in general, stomach pain when anxious, dull ache in the upper abdomen and heartburn)
Kanoni et al. [17]	98 patients with obesity (BMI ≥ 30 kg/m^2^) and NAFLD and randomized to either mastic 2.1 g/day or placebo for 6 months	- Improvement in total antioxidant status of NAFLD pts - interaction of mastic with cytokines and antioxidant biomarkers implicated in NAFLD pathogenesis
Moudi et al. [18]	147 postpartum women randomized to topical application of 15 g mastic for 3 days on episiotomy wound or to placebo	- Higher healing rates of episiotomy wound - no effect on episiotomy pain
Triantafyllou et al. [19]	133 subjects were randomized to either 5 g mastic powder (high dose) or mastic solution for 18 months	- Decrease in serum total cholesterol, LDL, total lipoprotein (a), apolipoprotein A-1, apolipoprotein B, SGOT, SGPT and γ-GT levels
Kartalis et al. [20]	156 subjects received different doses of mastic for 8 weeks	- Reduction in TC in subjects receiving crude mastic 1 g/day (highest dose) - no effect on LDL, HDL, triglycerides, uric acid and CRP
Kontogiannis et al. [21]	27 subject (13 hypertensive) randomized to receive one dose of 2.8 g mastic	- Acute decrease in peripheral and aortic SBP in hypertensive pts - no changes in normotensive pts
Kottakis et al. [22]	5 pts with H. Pylori infection and 3 controls treated with 1 g of mastic daily for 2 months	- Mastic’s arabinogalactan proteins inhibit neutrophil activation in the presence of H. Pylori neutrophil activating protein
Dabos et al. [23]	52 pts with H. Pylori randomized to receive either 350 mg tid of mastic for 14 days (Group A), or 1.05 g tid of mastic (Group B) for 14 days, or pantoprazole 20 mg bd plus mastic 350 mg tid for 14 days (Group C) or pantoprazole 20 mg bd plus amoxicillin 1 g bd plus clarithromycin 500 mg bd for 10 days (Group D)	- Eradication of H. pylori was confirmed in 4/13 pts in Group A, in 5/13 in Group B, in 0/13 in Group C and in 10/13 in Group D
Bebb et al. [24]	8 pts with H. Pylori 1 g mastic four times daily for 14 days	- No effect on H. Pylori status

Pts: patients; CD: Crohn’s disease; IL-6: interleukin-6; CRP: C-reactive protein; TNF-a: tumor necrosis factor-alpha; IBD: inflammatory bowel disease; IBDQ: IBD questionnaire; oxLDL: oxidized LDL; TC: total cholesterol; SBP: systolic blood pressure; tid: three times a day; bd: twice daily; NAFLD: non-alcoholic fatty liver disease.

## Data Availability

Not applicable.
